# Down‐regulation of interferon regulatory factor 2 binding protein 2 suppresses gastric cancer progression by negatively regulating connective tissue growth factor

**DOI:** 10.1111/jcmm.14677

**Published:** 2019-09-27

**Authors:** Yangyang Yao, Yi Wang, Li Li, Xiaojun Xiang, Junhe Li, Jun Chen, Zhen Liu, Shanshan Huang, Jianping Xiong, Jun Deng

**Affiliations:** ^1^ Department of Oncology The First Affiliated Hospital of Nanchang University Nanchang Jiangxi Province China; ^2^ Radiotherapy＆Chemotherapy Department HwaMei Hospital University of Chinese Academy of Sciences Ningbo Zhejiang Province China

**Keywords:** connective tissue growth factor, gastric cancer, IRF2BP2, prognosis

## Abstract

Interferon regulatory factor 2 binding protein 2 (IRF2BP2) is a transcriptional repressor involved in regulating gene expression and other biological processes, including tumorigenesis. However, the clinical significance and roles of IRF2BP2 in human gastric cancer (GC) remain uncertain. Clinical GC tissues were obtained from GC patients at the First Affiliated Hospital of Nanchang University. Immunohistochemistry (IHC) was conducted to detect the IRF2BP2 protein in clinical paraffin specimens. Cell proliferation, migration and invasion were evaluated by MTT, colony formation assays and transwell assays. Co‐immunoprecipitation was conducted to detect the interaction between TEA domain family members 4 (TEAD4) and vestigial‐like family member 4 (VGLL4) or Yes‐associated protein 1 (YAP1). Dual‐luciferase reporter assay was used to confirm the binding of miR‐101‐3p to the 3′‐UTR. The expression of IRF2BP2 was significantly higher in GC tissues than in normal tissues. Patients with higher IRF2BP2 protein expression had lower survival. IRF2BP2 knockdown inhibited proliferation, migration, invasion and epithelial‐mesenchymal transition in GC cells. IRF2BP2 knockdown decreased the mRNA and protein levels of connective tissue growth factor (CTGF). The interaction between IRF2BP2 and VGLL4 increased the binding of TEAD4 to YAP1, resulting in the transcriptional coactivation of CTGF. In addition, miR‐101‐3p suppressed the expression of CTGF by directly targeting the 3′‐UTR of IRF2BP2. Taken together, these findings provide a model for the role of miR‐101‐3p‐IRF2BP2‐CTGF signalling axis in GC and a novel insight into the mechanism of GC progression and metastasis.

## 1 INTRODUCTION

Gastric cancer (GC) is one of the most prevalent gastrointestinal malignancies, and mortality from GC is the third leading cause of cancer‐related deaths worldwide.^1^ The incidence of GC is highest in eastern Asia, and approximately 42% of cases occurred in China.^2^ Early diagnosis of GC is challenging, and most patients are diagnosed at an advanced stage. Despite tremendous advances in surgery, chemotherapy, radiotherapy and targeted molecular therapy, the overall effectiveness of treatment is low, with the 5‐year survival rate being <35%.^3^ Furthermore, the median overall survival (OS) of GC is currently <12 months.^4^ Therefore, exploring new diagnostic and prognostic markers is essential for developing targeted therapies for GC.

The interferon regulatory factor 2 binding protein 2 (IRF2BP2) gene encodes a nuclear protein that contains an N‐terminal zinc finger and a C‐terminal RING finger domain of the C3HC4 subclass that interacts with the C‐terminal transcriptional repression domain of interferon regulatory factor 2 (IRF2), which is a class of transcription factors that can regulate interferon expression.^5^, ^6^, ^7^ IRF2BP2 is an IRF2‐dependent transcriptional corepressor that can inhibit both enhancer‐activated transcription and baseline transcription, and inhibition is not mediated by histone deacetylase activity.^8^ IRF2BP2 also performs IRF‐2–independent functions, including the negative regulation of the nuclear factor of activated T cell 1 (NFAT1)–mediated transactivation of NFAT‐responsive promoters, consequently affecting the cell cycle, differentiation and apoptosis.^9^ IRF2BP2 is regulated by cancer‐related molecules. Koeppel et al^10^ have shown that IRF2BP2 is a transcriptional target of p53, promotes the proliferation of U2OS cells and accelerates cellular resistance to apoptosis induced by doxorubicin and actinomycin D treatment by inhibiting the p53‐mediated transactivation of the P21 and BAX genes. Whole transcriptome sequencing identified a novel IRF2BP2‐caudal type homeobox 1 fusion protein in mesenchymal chondrosarcoma and an IRF2BP2‐retinoic acid receptor alpha fusion protein in acute promyelocytic leukaemia.^11^, ^12^ These studies suggest that IRF2BP2 may play a role in tumorigenesis and cancer progression.

Connective tissue growth factor (CTGF) is a matricellular protein of the cysteine‐rich angiogenic inducer 61 (Cyr61)/CTGF/Nov family involved in many physiological and pathological processes, including carcinogenesis and regulation of the tumour microenvironment. In GC patients, elevated CTGF expression is strongly correlated with lymph node metastases, peritoneal dissemination and poor prognosis.^13^, ^14^, ^15^, ^16^ Moreover, CTGF expression levels are positively associated with the levels of vascular endothelial growth factors C and D (VEGF‐C and VEGF‐D).^13^ A study suggests that CTGF promotes GC cell proliferation by inducing the expression of cyclin D1,^16^ and the down‐regulation of CTGF inhibits GC cell metastasis and decreases the expression and proteolytic activity of both matrix metalloproteinase (MMP)‐2 and MMP‐9.^15^ CTGF binds to multiple cell surface receptors in a context‐dependent manner and functions as an oncogene in different types of tumours.^17^, ^18^, ^19^ CTGF is also one of the target genes downstream of the transcriptional coactivator Yes‐associated protein 1 (YAP1), which is an essential effector of the Hippo pathway. YAP1 accumulates in the nucleus, where it binds primarily to DNA‐binding transcription factors TEA domain family members 1‐4 (TEAD1‐4) and transcriptionally coactivates CTGF, contributing to tumorigenesis and cancer progression.^20^, ^21^ VGLL4 serves as a transcriptional corepressor in the nucleus by binding to TEAD4 and blocking transcriptional coactivation; moreover, VGLL4 competes with YAP1 for binding to TEAD4.^21^, ^22^

A previous study indicates that VGLL4 binds to IRF2BP2 to promote PD‐L1 expression and induces immune evasion through IRF2 inhibition in lung cancer.^23^ The binding of VGLL4 to IRF2BP2 also activates the expression of VEGF‐A in muscle cells.^24^ This evidence implies that IRF2BP2 may cross talk with Hippo pathway and CTGF via binding to VGLL4.

The Cancer Genome Atlas (TCGA) database shows the amplification of the IRF2BP2 gene in the majority of malignant tumours, including GC, which prompted us to focus on the clinical significance and roles of IRF2BP2 in human GC. This study evaluated the effect of the overexpression of IRF2BP2 in GC, indicating that IRF2BP2 regulated the expression of CTGF in a YAP1‐dependent manner and that the miR‐101‐3p‐IRF2BP2‐CTGF axis could be a potential prognostic marker and therapeutic target in GC.

## 2 MATERIALS AND METHODS

### 2.1 Patients and clinical specimens

Paraffin‐embedded GC tissue samples (n = 65) and adjacent noncancerous gastric tissues (n = 20) were from patients who underwent resection at the First Affiliated Hospital of Nanchang University between January 2009 and December 2011. Complete clinicopathological data of all the patients were available (Table 1). Fresh GC tissues (n = 40) and paired adjacent noncancerous tissues were stored in liquid nitrogen before use (Table S2). All patients agreed to participate in the study and provided written informed consent.

**Table 1:** Association of IRF2BP2 expression with clinicopathological parameters of gastric cancer patients

Parameters	IRF2BP2 expression (n = 65)			*P* value
	High	Low	Total	
Gender				
Male	15	21	36	**.053**
Female	19	10	29	
Age (y)				
≤60	26	16	42	**.036**
>60	8	15	23	
Histological grade				
Low	13	13	26	**.761**
Moderate, or high	21	18	39	
Tumour size (cm)				
≤4	16	27	43	**.0006**
>4	18	4	22	
TNM stage				
I + II	11	22	33	**.0002**
III + IV	23	9	32	
pT stage				
pT1 + pT2	9	20	29	**.0002**
pT3 + pT4	25	11	36	
Lymph node status				
N0 + N1	8	24	32	**.0008**
N2 + N3	26	7	33	
Perineural invasion				
No	14	21	35	**.03**
Yes	20	10	30	
Vascular invasion				
No	24	22	46	**.802**
Yes	10	9	19	
Total	34	31	65	

### 2.2 Cell lines and culture

Human embryonic kidney cell line HEK293T, human GC cell lines MKN‐45, AGS, HGC‐27, BGC‐823, SGC‐7901 and MGC‐803, and the human immortalized gastric epithelial cell line GES‐1 were obtained from the Cell Bank of the Chinese Academy of Sciences (Shanghai, China). The cells were cultured in RPMI 1640 or Dulbecco’s modified Eagle’s medium (Solarbio) containing 10% foetal bovine serum (Transgene) at 37°C with 5% CO_2_.

### 2.3 Immunohistochemistry

Immunohistochemistry was performed as previously described.^25^ Slides were incubated with IRF2BP2 rabbit polyclonal antibody (1:100; Abcam, ab180891) in a humidified chamber overnight at 4°C, washed thrice with PBS and incubated with secondary antibody for 50 min at 37°C. Sections were washed, developed with 3,3′‐diaminobenzidine tetrahydrochloride and counterstained with haematoxylin before mounting. All scores were evaluated by two pathologists who were blinded to the pathological information. Immunohistochemical grading standards were performed according to the previously described methods.^25^

### 2.4 Cell transfection

Small interfering RNA (siRNA) targeting IRF2BP2 (IRF2BP2 siRNA‐1/2/3) and a negative control (NC) siRNA were purchased from GenePharma (Shanghai) and consisted of the following sequences: IRF2BP2 siRNA‐1, 5′‐GCCCUUCGAGAGCAAGUUUTT‐3′; IRF2BP2 siRNA‐2, 5′‐CCCUGAUCUUAGUAGCAGATT‐3′; and IRF2BP2 siRNA‐3, 5′‐CCGUCCUCUAUGAACCAAATT‐3′. miRNA mimics were obtained from GenePharma (Shanghai) and comprised the following sequences: miR‐101‐3p, 5′‐UACAGUACUGUGAUAACUGAA‐3′ (sense) and 5′‐CAGUUAUCACAGUACUGUAUU‐3′ (antisense); miR‐30a‐5p, 5′‐UGUAAACAUCCUCGACUGGAAG‐3′ (sense) and 5′‐UCCAGUCGAGGAUGUUUACAUU‐3′ (antisense); miR‐519d‐3p, 5′‐CAAAGUGCCUCCCUUUAGAGUG‐3′ (sense) and 5′‐CUCUAAAGGGAGGCACUUUGUU‐3′ (antisense); miR‐155‐5p, 5′‐UUAAUGCUAAUCGUGAUAGGGGUU‐3′ (sense) and 5′‐CCCCUAUCACGAUUAGCAUUAAUU‐3′ (antisense); and NC, 5′‐UUCUCCGAACGUGUCACGUTT‐3′ (sense) and 5′‐ACGUGACACGUUCGGAGAATT‐3′ (antisense). Cells were grown to a confluence of 30%‐50% and transfected using Lipofectamine 2000 (Invitrogen).

### 2.5 Immunoblotting

The proteins were extracted from GC tissues or cells by lysis buffer (10 mmol/L Tris pH 7.4, 2% SDS). Equal amounts of protein were separated by SDS‐PAGE. Proteins were detected with anti‐IRF2BP2 (1:1000; Proteintech, 18847‐1‐AP), β‐actin (1:2000; Cell Signaling Technology, #4967), E‐cadherin (1:1000; Cell Signaling Technology, #14472), N‐cadherin (1:1000; Cell Signaling Technology, #13116), vimentin (1:1000; Cell Signaling Technology, #5741), VGLL4 (1:1000; Abcam, ab140290), YAP1 (1:1000; CST, 14074s) or CTGF (1:1000; Cell Signaling Technology, #86641).

### 2.6 RNA extraction and real‐time quantitative PCR

Total RNA from GC cell lines and tissues was extracted using TRIzol reagent (Invitrogen) and reverse‐transcribed to cDNA using the EasyScript First‐Strand cDNA Synthesis SuperMix Kit (TransGen Biotech). Real‐time quantitative PCR (RT‐qPCR) was performed with the StepOnePlus Real‐Time PCR System (Applied Biosystems) and Fast Start Universal SYBR Green Master Mix (Takara). The relative expression of IRF2BP2 (forward, 5′‐AGGTTGTTGGGTTTCGAGGC‐3′; reverse, 5′‐GGCGGAGACACAAAAGAGGA‐3′) and CTGF (forward, 5′‐GTTTGGCCCAGACCCAACTA‐3′; reverse, 5′‐GGCTCTGCTTCTCTAGCCTG‐3′) relative to the internal control (GAPDH) was analysed using the $2^{-\Delta \Delta C_{T}}$ method.

### 2.7 Cell proliferation and colony formation assays

Forty‐eight hours after transfection, SGC‐7901 or BGC‐823 cells were plated in 96‐well plates at a density of 2000 cells/well for MTT assays or seeded in six‐well plates at a density of 300 cells/well for colony formation assays following the methods described previously.^25^

### 2.8 Migration and invasion assays

Cell invasion and migration were conducted using 8‐µm transwell inserts (Costar) coated with or without 60 µL of Matrigel (BD Biosciences), which were placed into each well of a 24‐well plate. Forty‐eight hours after transfection, SGC‐7901 and BGC‐823 cells (density 2 × 10^4^) in 200 µL of serum‐free medium were transferred to the upper chamber following the methods previously described.^26^ Cells were stained with crystal violet and counted by direct microscopic visualization.

### 2.9 Co‐immunoprecipitation

The cell lysates were centrifuged, and the supernatants were transferred to test tubes. The IRF2BP2 antibody (Proteintech, 18847‐1‐AP), TEAD4 antibody (Proteintech 12418‐1‐AP) or IgG (served as a control) was incubated with the corresponding supernatant at 4°C for 4 hours. After that, protein A/G beads (Santa Cruz Biotechnology) were added to the tubes, and the mixtures were incubated at 4°C for 2 hours. Beads were washed thrice with lysis buffer, and Western blotting was performed to detect the bound proteins.

### 2.10 Vector construction and dual‐luciferase reporter assay

The present study predicted the binding of miR‐101‐3p to the 3′‐UTR of IRF2BP2. The 3′‐UTR sequence containing the miR‐101‐3p (wild‐type) binding site or the mutated binding site was amplified by GenePharma (Shanghai). Reporter plasmids were obtained by gene splicing and overlap extension PCR (SOEPCR). For the dual‐luciferase reporter assay, HEK293T cells were plated into 24‐well plates and transfected with reporter plasmids using Lipofectamine 2000 (Invitrogen). Six hours after transfection, the cells were washed with D‐Hanks solution, then cultured for 18 hours, and luciferase activity was detected using the Dual‐Luciferase Reporter Assay System (Promega, E1960). The effects of miR‐101‐3p on the wild‐type and mutated IRF2BP2 3′‐UTR were assessed by measuring firefly luciferase activity normalized to Renilla luciferase activity.

### 2.11 Xenografted tumour model and staining

BALB/c‐nu mice (5‐6 weeks old) were purchased from the SLACCAS Experimental Animal Company (Shanghai). The mice were randomly divided into two groups (six animals per group). For the subcutaneous assay, 1 × 10^7^ SGC‐7901 cells that transfected with lentivirus encoding IRF2BP2 shRNAs or scramble shRNA were subcutaneously injected. The tumour volume was measured every 3 days with a calliper and calculated using the formula (L × W^2^)/2, where L is the length diameter and W is the width diameter of the tumour. After 21 days, the tumours were excised. All mice were handled according to the institutional guidelines.

### 2.12 Statistical analysis

Data were analysed using SPSS software version 20.0 (SPSS). The difference between the two groups was determined by Student's *t* test. Survival curves were plotted using the Kaplan‐Meier method and compared by the log‐rank test. Correlations between IRF2BP2 mRNA and CTGF mRNA expression were estimated using the Spearman correlation coefficient. *P* values <.05 were considered statistically significant.

## 3 RESULTS

### 3.1 IRF2BP2 expression is up‐regulated in GC cell lines and tissues

**Figure 1:**
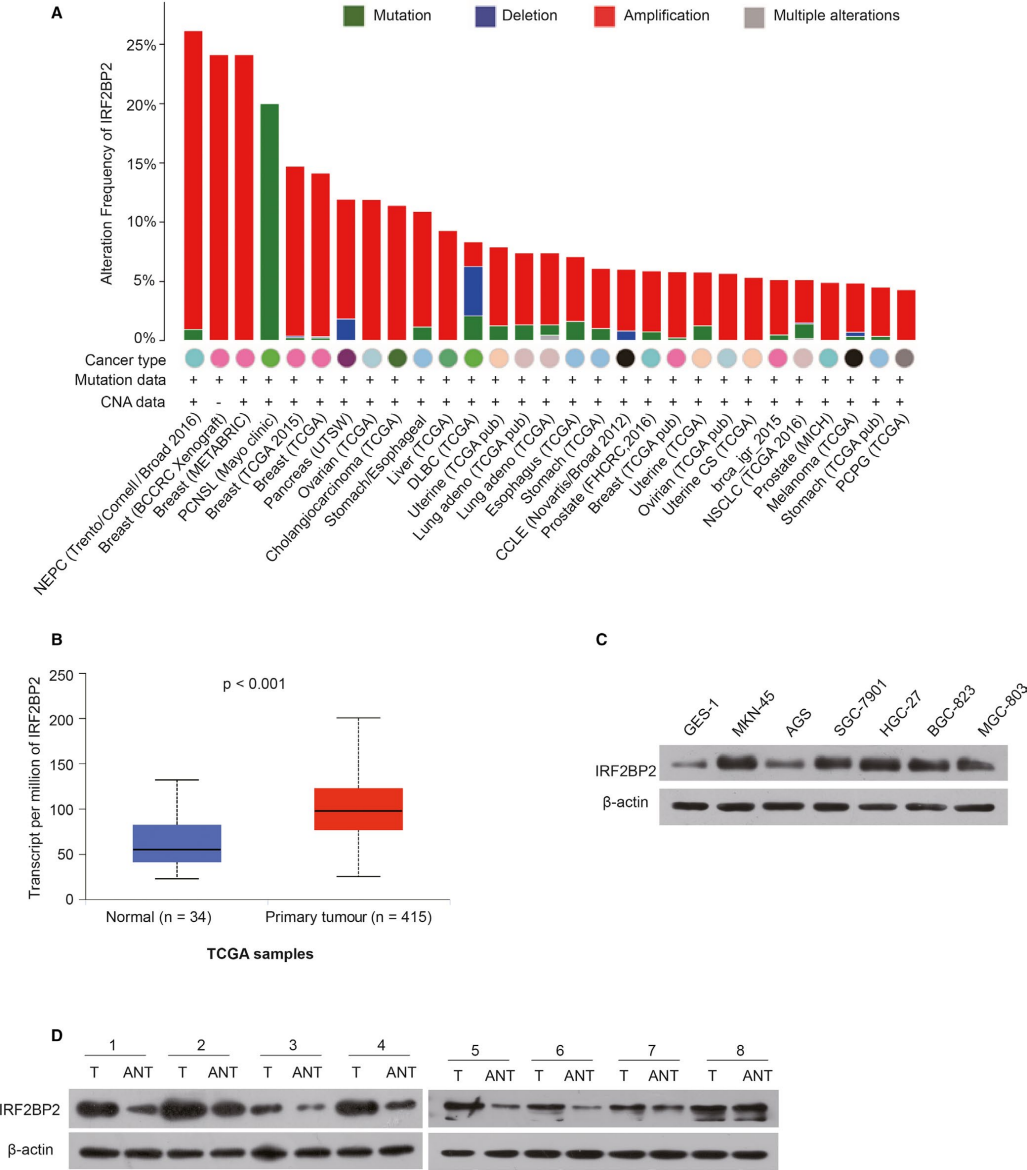
IRF2BP2 expression is up‐regulated in GC. A, An analysis of the database in cBioPortal showed that IRF2BP2 was amplified in most tumours. B, The TCGA database indicated that the expression of IRF2BP2 mRNA was significantly higher in primary GC tissues (n = 415) than in normal tissues (n = 34) (*P* < .001). C, The IRF2BP2 protein levels were higher in GC cell lines than in the GES‐1 cell line. D, IRF2BP2 protein expression was higher in GC tissues than in adjacent noncancerous gastric tissues. GC, gastric cancer; IRF2BP2, interferon regulatory factor 2 binding protein 2; TCGA, The Cancer Genome Atlas

The bioinformatic analysis of the TCGA database indicated that IRF2BP2 was amplified in most types of tumours, including GC (http://www.cbioportal.org/) (Figure 1A), and the level of IRF2BP2 mRNA in primary GC tissues was significantly higher than that in normal tissues (http://ualcan.path.uab.edu/cgi-bin/TCGAExResultNew2.pl?genenam=IRF2BP2&ctype=STAD) (Figure 1B). Western blotting was used to determine the expression of IRF2BP2 in GC tissues and cell lines. The IRF2BP2 protein levels were significantly higher in all GC cell lines than in the human immortalized gastric epithelial cell line GES‐1 (Figure 1C). Moreover, IRF2BP2 protein expression was elevated in GC tissues relative to adjacent noncancerous gastric tissues (n = 8) (Figure 1D).

### 3.2 Predicted prognostic value of IRF2BP2 in GC patients

**Figure 2:**
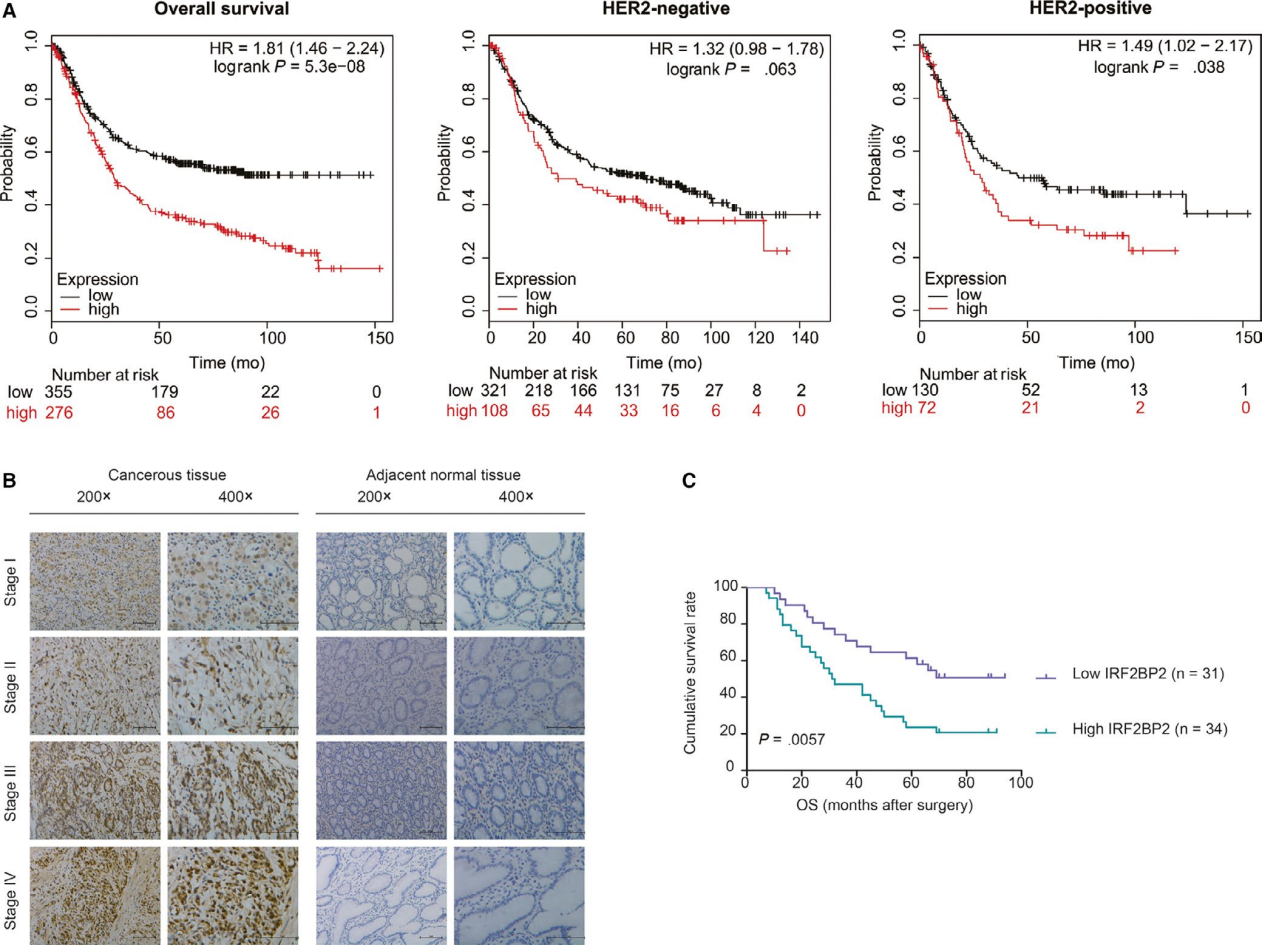
The predicted prognostic value of IRF2BP2 in GC patients. A, The Km‐plot survival database predicted an association between high IRF2BP2 mRNA levels and poor survival in GC, except in the HER2‐negative subgroup. B, Representative IHC staining of the IRF2BP2 protein in GC tissues and adjacent noncancerous gastric tissues (scale bar: 100 μm). C, The overall survival rate of GC was worse in patients with high IRF2BP2 than in patients with low IRF2BP2 (n = 65, *P* < .01). GC, gastric cancer; IRF2BP2, interferon regulatory factor 2 binding protein 2

Based on the Km‐plot database (https://www.Kmplot.com), the prognostic value of IRF2BP2 mRNA expression was linked to worse OS in GC patients (n = 631, –HR = 1.81 [1.46‐2.24], *P* = 5.3e‐08) (Figure 2A). The Affymetrix ID was valid: 224572‐s‐at (IRF2BP2). Since human epidermal growth factor receptor 2 (HER2) is an important marker in GC,^27^ we examined the predicted prognostic value of IRF2BP2 in the HER2‐positive and HER2‐negative subgroups. The results suggested that high IRF2BP2 mRNA levels were linked with worse OS in HER2‐positive patients (n = 202, –HR = 1.49 [1.02‐2.17], *P* = .038) but not in HER2‐negative patients (n = 429, –HR = 1.32 [0.98‐1.78], *P* = .063) (Figure 2A).

To further confirm the prognostic value of IRF2BP2 proteins, IHC was used to detect the expression of these proteins in GC tissues (n = 65). The results showed that IRF2BP2 was predominantly localized to the nucleus, IRF2BP2 protein expression was higher in GC tissues, and the TNM stage was positively correlated with the expression level of IRF2BP2; in contrast, IRF2BP2 expression was lower in adjacent noncancerous gastric tissues (Figure 2B). Next, the relationship between IRF2BP2 protein expression and the clinicopathological characteristics of GC was evaluated. IRF2BP2 expression was closely associated with age (*P* = .036), tumour size (*P* = .0006), TNM stage (*P* = .0002), depth of invasion (*P* = .0002), lymph node metastasis (*P* = .0008) and vascular invasion (*P* = .03) (Table 1). Furthermore, the clinical follow‐up data for patients with GC indicated that high IRF2BP2 expression contributed to poor OS (*P* = .0057) (Figure 2C), and the 5‐year survival rate was comparatively higher in patients with lower IRF2BP2 expression (Table S1).

### 3.3 Knockdown of IRF2BP2 inhibits GC cell proliferation and invasion

**Figure 3:**
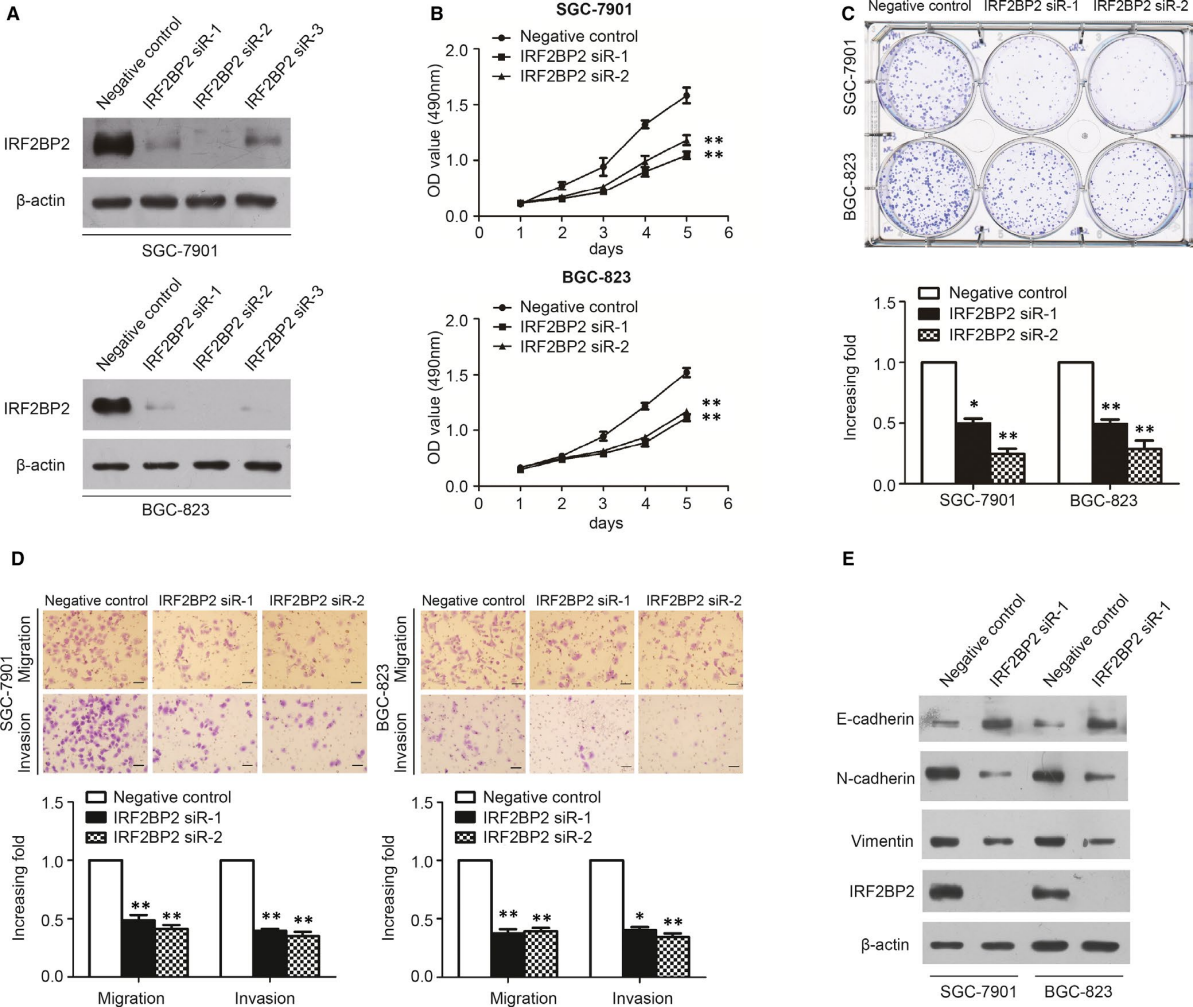
IRF2BP2 Knockdown inhibits cancer aggressiveness and EMT in GC. A, Western blotting was used to detect the transfection efficiency. B, MTT assay results showed that IRF2BP2 knockdown significantly suppressed the proliferation rates of SGC‐7901 and BGC‐823 cell lines. C, The knockdown of IRF2BP2 significantly suppressed colony formation in the SGC‐7901 and BGC‐823 cell lines. D, IRF2BP2 knockdown significantly suppressed the migration and invasion of SGC‐7901 and BGC‐823 cell lines (scale bar: 50 μm). E, IRF2BP2 knockdown increased E‐cadherin expression, and inhibited N‐cadherin and vimentin protein expression in the SGC‐7901 and BGC‐823 cell lines. *P < .05, **P < .01, data were expressed as the mean ± SEM. GC, gastric cancer; IRF2BP2, interferon regulatory factor 2 binding protein 2

To further investigate the function of IRF2BP2, IRF2BP2 expression in the SGC‐7901 and BGC‐823 cell lines was inhibited by siRNAs (NC siRNA and IRF2BP2 siRNA‐1/2/3, respectively). Western blotting was used to determine the transfection efficiency, and the results showed that IRF2BP2 expression decreased significantly in IRF2BP2 siRNA‐1/2/3–transfected cells relative to NC siRNA‐transfected cells (Figure 3A).

The effects of IRF2BP2 on GC cell proliferation were analysed by MTT and colony formation assays. The results suggested that the proliferation rate and colony formation ability were significantly decreased in the IRF2BP2 siRNA‐transfected group compared to the NC group (Figure 3B,C).

Given that IRF2BP2 expression was correlated with lymph node metastasis and vascular invasion in clinical samples, a transwell assay was performed to investigate the role of IRF2BP2 in migration and invasion. The knockdown of IRF2BP2 significantly inhibited cell migration and invasion (Figure 3D). In addition, epithelial‐mesenchymal transition (EMT) markers were detected by Western blotting. IRF2BP2 knockdown up‐regulated E‐cadherin and down‐regulated N‐cadherin and vimentin (Figure 3E), indicating that IRF2BP2 knockdown suppresses the migration, invasion and proliferation of GC cells.

### 3.4 The binding of IRF2BP2 to VGLL4 increases the binding of TEAD4 to YAP1, leading to the transcriptional coactivation of CTGF expression

**Figure 4:**
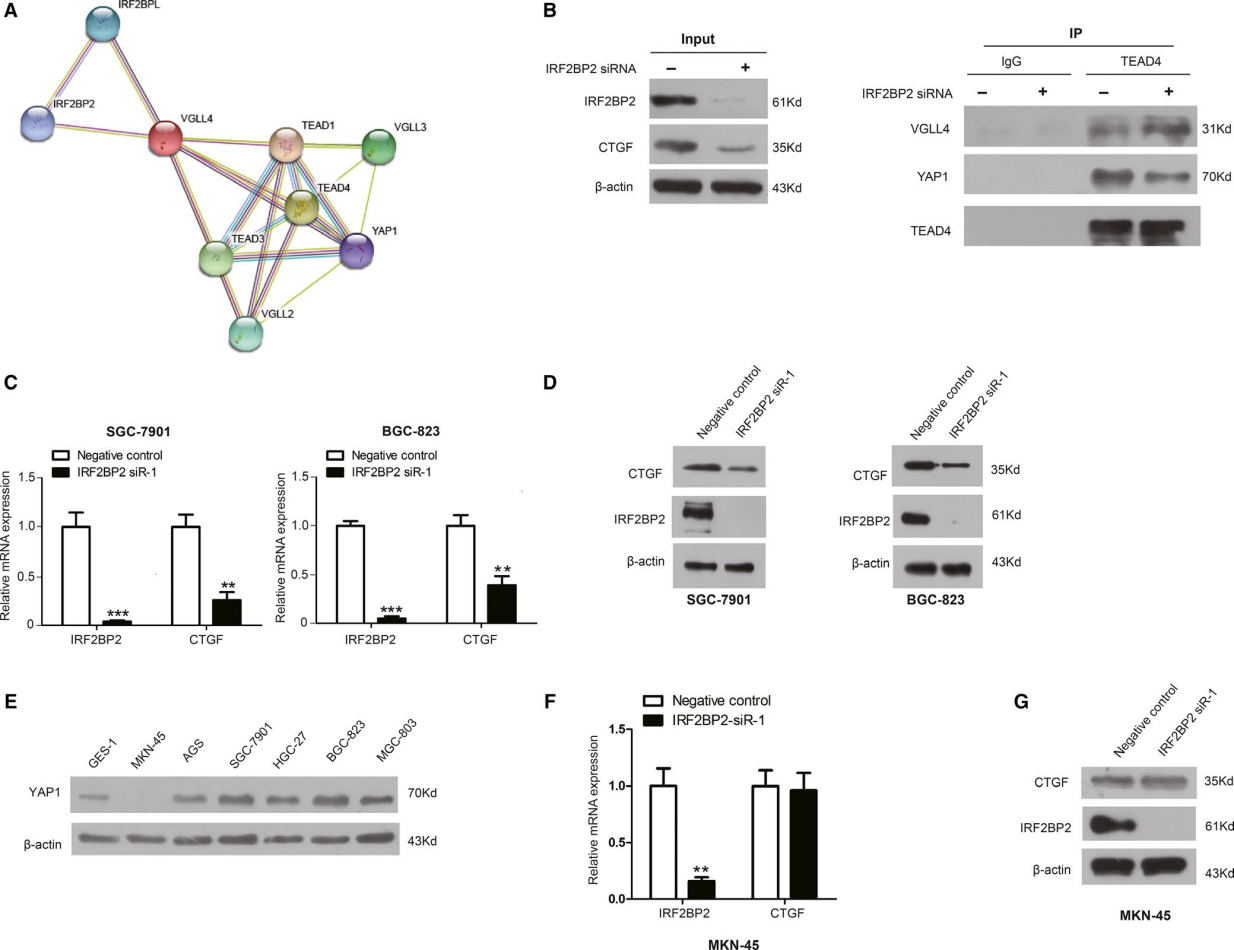
IRF2BP2 regulates CTGF expression by competing with TEAD4 for binding to VGLL4. A, The proteomic database STRING predicted that VGLL4 interacted with IRF2BP2. B, Co‐immunoprecipitation with anti‐TEAD4 antibody showed that IRF2BP2 knockdown increased the binding of TEAD4 to VGLL4 and decreased the binding of YAP1 to TEAD4. C, The results of RT‐qPCR indicated that down‐regulating IRF2BP2 decreased CTGF mRNA in the SGC‐7901 and BGC‐823 cell lines. D, The results of Western blotting demonstrated that IRF2BP2 knockdown decreased the CTGF protein level in GC cell lines SGC‐7901 and BGC‐823. E, Expression of YAP1 protein in GC cell lines. F, RT‐qPCR was performed in MKN‐45 cells and demonstrated that IRF2BP2 knockdown did not significantly affect CTGF mRNA expression. G, The results of Western blotting in MKN‐45 cells revealed that IRF2BP2 knockdown did not significantly affect CTGF protein expression. **P < .01, ***P < .001, data were expressed as the mean ± SEM. CTGF, connective tissue growth factor; GC, gastric cancer; IRF2BP2, interferon regulatory factor 2 binding protein 2; RT‐qPCR, real‐time quantitative PCR; TEAD4, TEA domain family members 4; VGLL4, vestigial‐like family member 4

This study further explores the specific mechanism of function of IRF2BP2. Data from the proteomic database STRING (https://string-db.org/cgi/network.pl?taskId=gcaogyyOxVi0) predicted that IRF2BP2 could interact with VGLL4 (Figure 4A). Moreover, studies proved that the binding of IRF2BP2 to VGLL4 played an important role in immune evasion and angiogenesis.^23^, ^24^ It has been established that VGLL4 is a transcriptional corepressor that competes with the transcriptional coactivator YAP1 for binding to TEAD4.^21^, ^22^ To assess whether IRF2BP2 affects the binding of YAP1 to TEAD4, IRF2BP2 was knocked down in SGC‐7901 cells, and co‐immunoprecipitation was performed with anti‐TEAD4 antibody. The Western blot results revealed that IRF2BP2 knockdown increased the binding of TEAD4 to VGLL4 and decreased the binding of TEAD4 to YAP1 (Figure 4B).

Connective tissue growth factor is a critical gene downstream of YAP1 and has been shown to promote the proliferation and invasion of GC cells. The increased expression of CTGF in human GC samples is correlated with lymph node metastases, peritoneal dissemination and poor prognosis.^13^, ^14^, ^15^, ^16^, ^28^ Considering the important role of CTGF in GC, RT‐qPCR and Western blot were conducted to confirm the relationship between IRF2BP2 and CTGF. The results indicated that the knockdown of IRF2BP2 significantly decreased the expression of CTGF at both the mRNA and protein levels in the SGC‐7901 and BGC‐823 cell lines (Figure 4C,D).

Since an intragenic homozygous deletion, the expression of YAP1 was completely lost in the GC cell line MKN‐45^29^ (Figure 4E). The MKN‐45 cell line was used to determine whether IRF2BP2 regulates CTGF through YAP1. IRF2BP2 was knocked down in MKN‐45 cells, and the results of RT‐qPCR and Western blot indicated that there were no significant changes in CTGF expression at both the mRNA and protein levels (Figure 4F,G).

### 3.5 The expression of CTGF and IRF2BP2 is positively correlated in clinical GC samples

**Figure 5:**
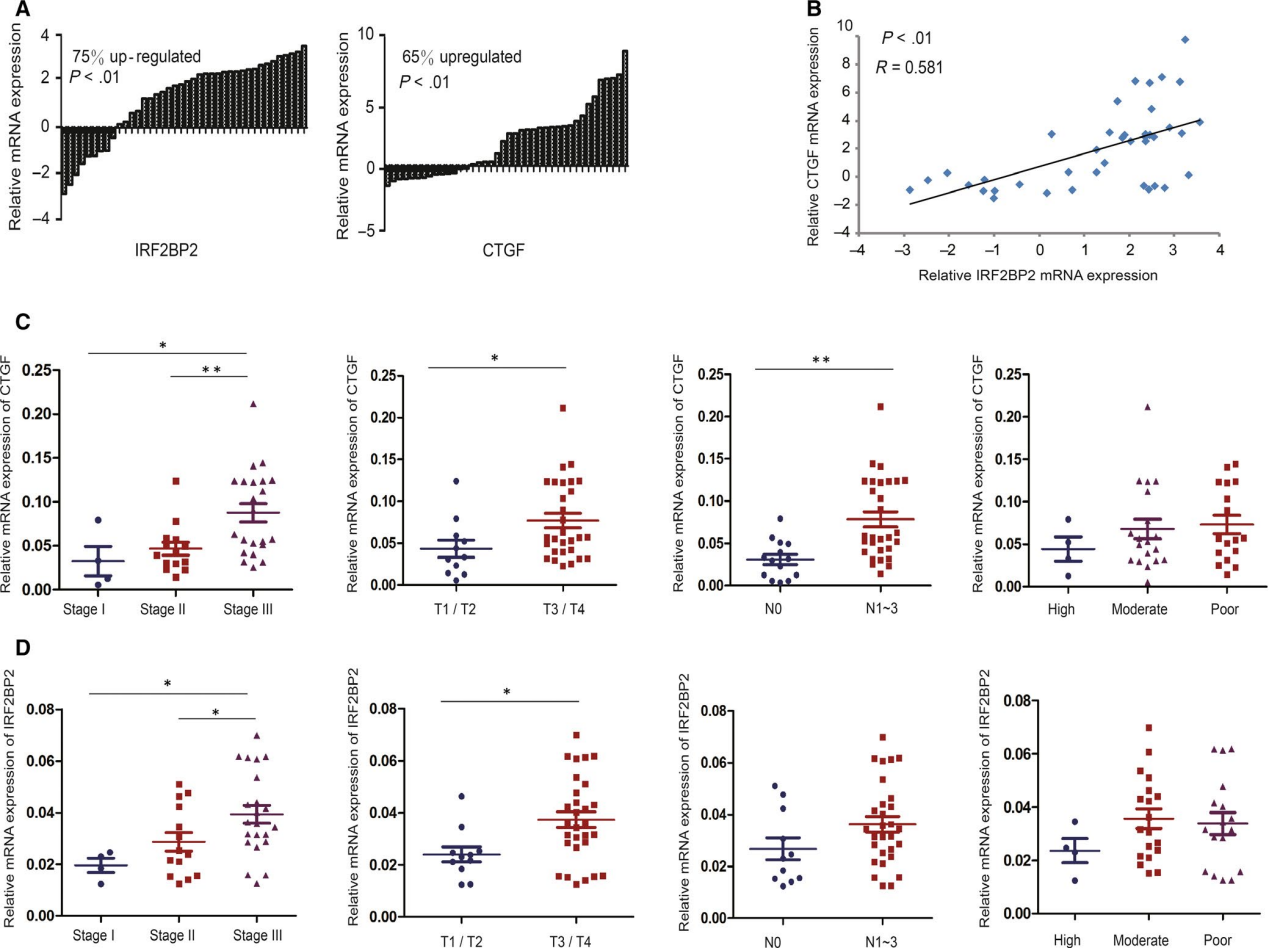
Expression of IRF2BP2 and CTGF mRNA in fresh GC samples. A, The results of RT‐qPCR showed that the mRNA levels of IRF2BP2 and CTGF were higher in 40 fresh GC samples than in the paired adjacent normal tissues. B, IRF2BP2 mRNA levels were positively correlated with CTGF mRNA levels in these 40 samples. C, Relationship between CTGF mRNA expression and tumour stage or differentiation. D, Relationship between IRF2BP2 mRNA expression and tumour stage or differentiation. *P < .05, **P < .01. CTGF, connective tissue growth factor; GC, gastric cancer; IRF2BP2, interferon regulatory factor 2 binding protein 2

In clinical samples, 40 pairs of fresh GC tissue and adjacent noncancerous tissues were used (Table S2). The mRNA levels of IRF2BP2 and CTGF were higher in GC tissues than in paired noncancerous tissues (Figure 5A). Moreover, the up‐regulation of IRF2BP2 mRNA was positively associated with the up‐regulation of CTGF mRNA (*R* = 0.581) (Figure 5B). We further analysed the relationship between the mRNA expression of CTGF or IRF2BP2 and clinicopathological features, including TNM stage, lymphatic metastasis, depth of invasion and differentiation. High CTGF mRNA expression was correlated with the depth of invasion and lymphatic metastasis, and CTGF mRNA expression in patients with TNM stage III was significantly higher than that in patients with TNM stage I or II (Figure 5C). In addition, IRF2BP2 mRNA expression was positively associated with the depth of invasion, and the level of IRF2BP2 mRNA expression in patients with TNM stage III was significantly higher than that in patients with lower TNM stage (Figure 5D).

### 3.6 MicroR‐101‐3p suppresses CTGF expression through IRF2BP2‐YAP1 axis

**Figure 6:**
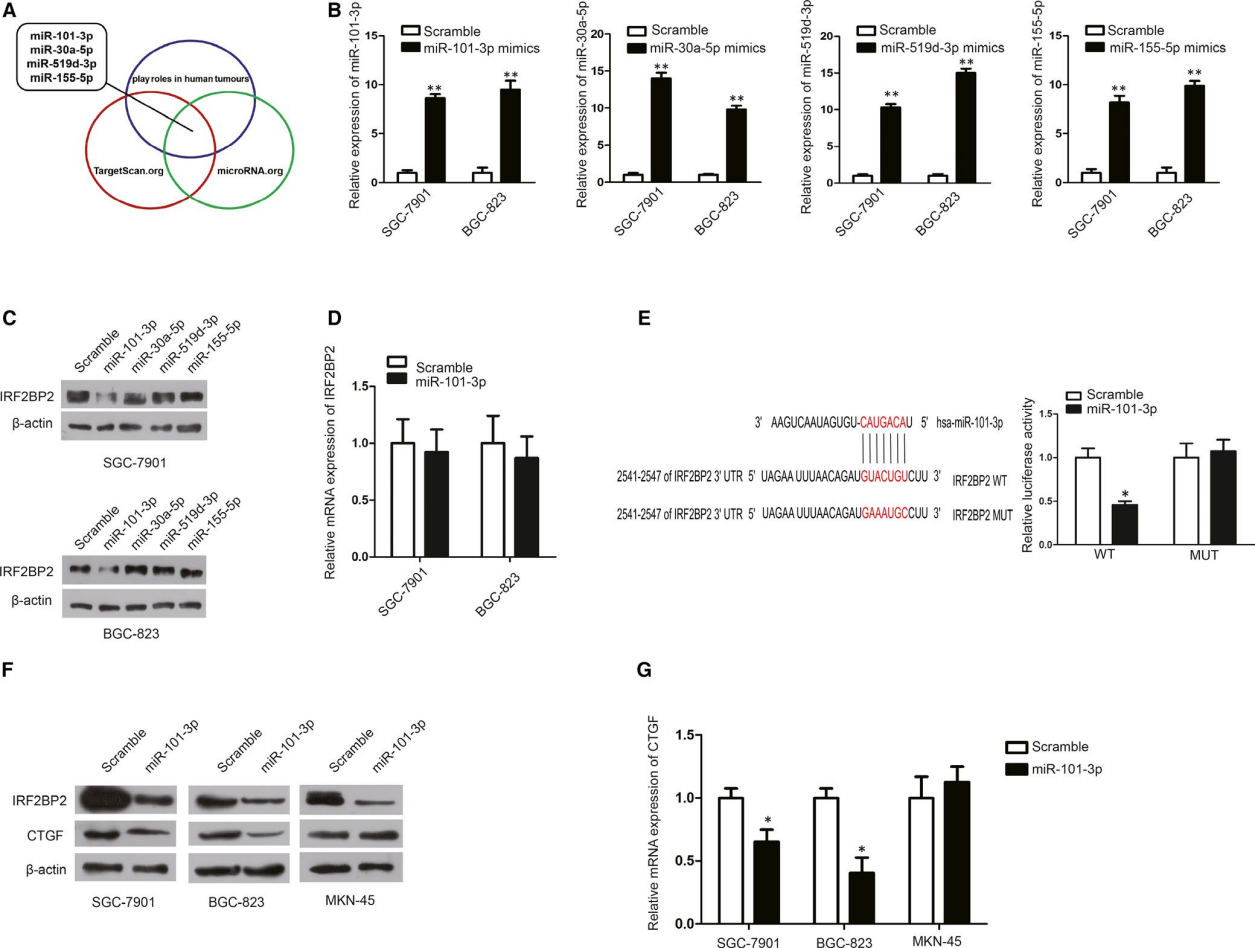
miR‐101‐3p targets IRF2BP2 directly. A, Through TargetScan.org and microRNA.org, four miRNAs, which have been reported to play roles in human tumours, were predicted to have a binding site on the 3′‐UTR of IRF2BP2. B, The expression of these miRNAs was increased in response to mimic transfection. C, The results of Western blotting showed that up‐regulating miR‐101‐3p significantly reduced the protein expression of IRF2BP2. D, miR‐101‐3p overexpression had no significant effect on IRF2BP2 mRNA levels. E, Luciferase assay results indicated that miR‐101‐3p overexpression suppressed luciferase activity from the wild‐type 3′‐UTR of IRF2BP2 but had no significant effect on the mutant‐type. F, miR‐101‐3p overexpression reduced the protein expression of both IRF2BP2 and CTGF in SGC‐7901 and BGC‐823 cells but only the expression of IRF2BP2 in MKN‐45 cells. G, miR‐101‐3p overexpression reduced the mRNA expression of CTGF in SGC‐7901 and BGC‐823 cells but not in MKN‐45 cells. *P < .05, **P < .01, data were expressed as the mean ± SEM. CTGF, connective tissue growth factor; IRF2BP2, interferon regulatory factor 2 binding protein 2

Considering the importance of miRNAs in transcriptional regulation,^30^, ^31^, ^32^ putative miRNAs that might regulate IRF2BP2 were identified by bioinformatic analysis. Several miRNAs with a predicted binding site in the 3′‐UTR of IRF2BP2 were identified using TargetScan (http://www.targetscan.org/) and microRNA.org (http://www.microrna.org/). Four miRNAs (miR‐101‐3p, miR‐30a‐5p, miR‐519d‐3p and miR‐155‐5p) reported to play roles in human tumours were chosen^33^, ^34^, ^35^, ^36^ (Figure 6A). The results of Western blotting in the SGC‐7901 and BGC‐823 cell lines showed that the overexpression of miR‐101‐3p significantly reduced IRF2BP2 protein expression (Figure 6B,C); however, the overexpression of miR‐101‐3p had no significant effect on IRF2BP2 mRNA expression (Figure 6D).

To determine whether miR‐101‐3p targets IRF2BP2 directly, plasmids were generated by cloning the wild‐type or mutant‐type 3'‐UTR of IRF2BP2. The results of luciferase assays in HEK293T cells showed that miR‐101‐3p transfection significantly suppressed the luciferase activity of the wild‐type 3'‐UTR of IRF2BP2 but not of the mutant‐type (Figure 6E).

To further investigate whether miR‐101‐3p regulates the expression of CTGF, miR‐101‐3p was overexpressed in SGC‐7901 cells, BGC‐823 cells and MKN‐45 cells. RT‐qPCR and Western blot results demonstrated that miR‐101‐3p overexpression inhibited CTGF mRNA and protein expression in both SGC‐7901 and BGC‐823 cells but not in MKN‐45 cells in which YAP1 was completely lost (Figure 6F,G), suggesting that miR‐101‐3p inhibited CTGF expression via the IRF2BP2‐YAP1 axis.

### 3.7 IRF2BP2 depletion impedes xenograft tumour growth

**Figure 7:**
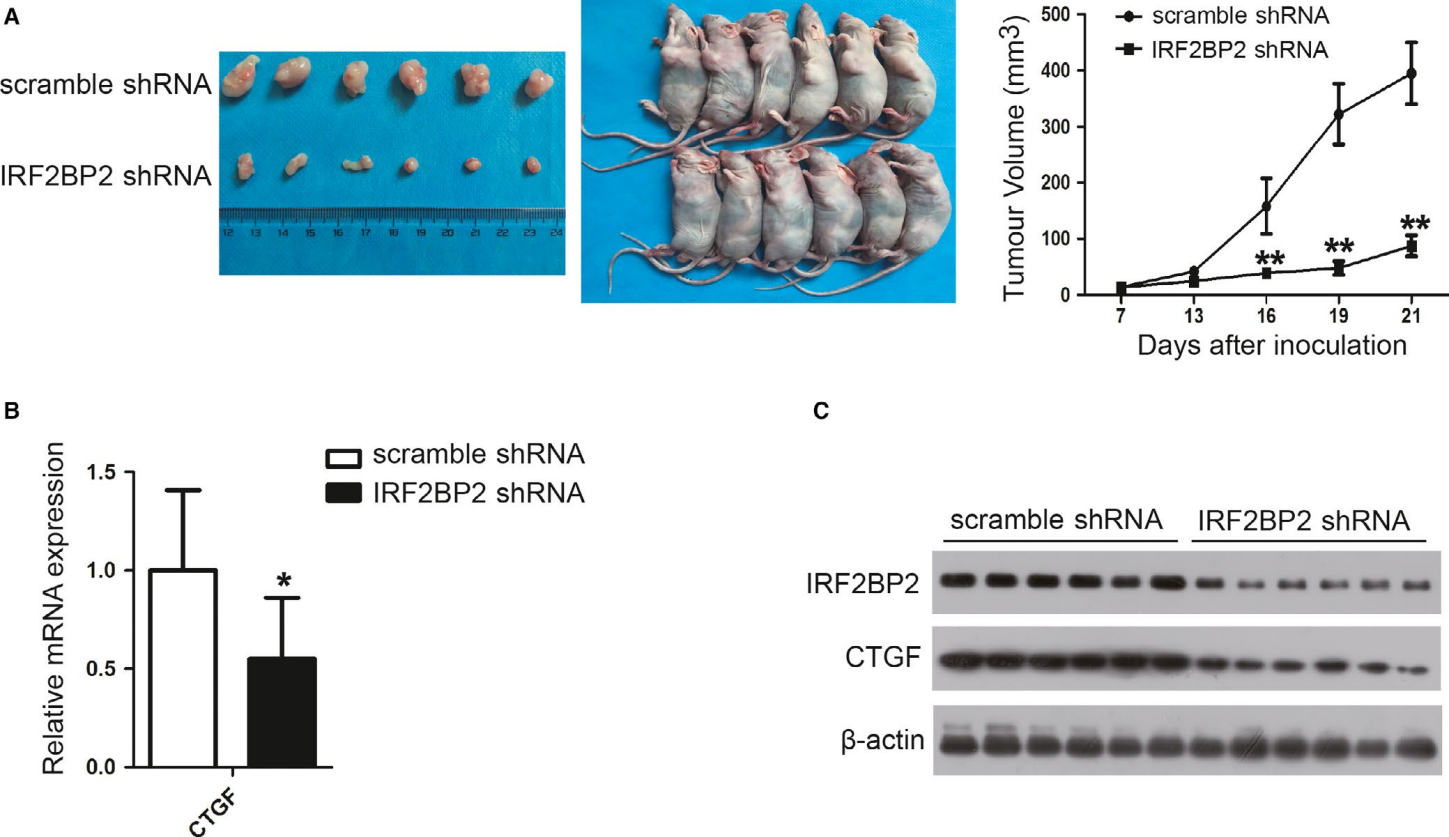
IRF2BP2 knockdown inhibits tumour growth in vivo. A, Xenograft tumours were harvested at the end of the experiment. After monitoring for 21 days, the growth curves of the xenograft tumours suggested that IRF2BP2 knockdown significantly inhibited tumour growth in vivo (mean ± SD, n = 6). B, The mRNA levels of CTGF were detected in six tumours by RT‐qPCR (mean ± SEM, n = 6). C, The protein levels of IRF2BP2 and CTGF were detected in six tumours by Western blotting. *P < .05, **P < .01. CTGF, connective tissue growth factor; GC, gastric cancer; IRF2BP2, interferon regulatory factor 2 binding protein 2; RT‐qPCR, real‐time quantitative PCR

To evaluate the biological significance of IRF2BP2, SGC‐7901 cells expressing scramble shRNA or IRF2BP2 shRNA were inoculated to build a xenograft mouse model. Tumour size was monitored for 21 days. The results suggest that the knockdown of IRF2BP2 significantly inhibits tumour growth (Figure 7A). To confirm the mechanisms identified in cell lines, the tumours were removed from mice and subjected to RT‐qPCR and Western blotting. Both the mRNA and protein levels of CTGF were significantly decreased upon IRF2BP2 knockdown, which is consistent with in vitro findings (Figure 7B,C).

## 4 DISCUSSION

IRF2BP2 has been reported to be involved in the malignancy of breast cancer, leukaemia and chondrosarcoma.^11^, ^12^, ^37^ The TCGA database revealed that the IRF2BP2 gene was amplified in most tumours, including GC. In addition, the level of IRF2BP2 mRNA was higher in primary GC tissues than in normal tissues. Therefore, our study focused on the roles and mechanisms of IRF2BP2 in GC.

In the present study, the IRF2BP2 protein expression levels were significantly increased in GC cell lines and tissues, indicating that IRF2BP2 was overexpressed in GC, potentially leading to the onset of GC. Furthermore, the Km‐plot database predicted that high IRF2BP2 mRNA levels were correlated with worse OS in GC patients. HER2 positivity is associated with pathogenesis and worse OS in several types of cancer, including GC.^38^ The Km‐plot database indicated that IRF2BP2 mRNA levels were linked with worse prognosis in HER2‐positive GC patients but not in HER2‐negative GC patients, and this characteristic may help select patients who will benefit from IRF2BP2‐targeted therapy. To confirm the clinical significance of IRF2BP2 shown in the Km‐plot database, IHC was used to detect the expression of IRF2BP2 protein in 65 GC samples and 20 samples of adjacent noncancerous gastric tissues. IRF2BP2 was primarily localized to the nucleus, which is consistent with studies suggesting the multiple functions of IRF2BP2 in the nucleus.^2^, ^3^, ^4^, ^5^, ^6^, ^7^, ^8^, ^9^ In addition, IRF2BP2 levels were positively and significantly correlated with tumour size, clinical stage, depth of invasion and lymph node metastasis. Survival analysis indicated that the 5‐year OS of patients with high IRF2BP2 expression was significantly lower than that of patients with low IRF2BP2 expression. Therefore, the IHC results suggest that the expression of IRF2BP2 is positively associated with tumour progression and poor prognosis in patients with GC.

The results in patient samples suggest that IRF2BP2 is involved in GC proliferation, migration and invasion. Then, the effects of IRF2BP2 expression on proliferation and invasion were assessed in GC cells. It has been reported that IRF2BP2 suppresses apoptosis and promotes cell proliferation in breast cancer cells.^39^ In the present study, MTT and colony formation assay results showed that knocking down IRF2BP2 significantly inhibited the proliferation of GC cells. In addition, migration and invasion are basic characteristics of malignant tumours^40^, ^41^ and the main cause of death in patients with GC, and a growing body of evidence indicates that tumour cells have high motility and invasiveness after EMT, in which E‐cadherin and vimentin are considered the most critical molecules.^42^ In the present study, knocking down IRF2BP2 inhibited the migration and invasion of GC cells and EMT.

We further explored how IRF2BP2 promotes proliferation, migration and invasion in GC cells. The proteomic database STRING predicted that IRF2BP2 could interact with VGLL4. Jiao et al^22^ demonstrated that VGLL4 had an antagonist function by directly competing with YAP1 for binding to TEADs, exhibiting potent antitumor activity against GC in vitro and in vivo. YAP1 is a major effector of the Hippo pathway. Activated YAP1 accumulates in the nucleus and induces a transcriptional programme critical for cell proliferation, migration and invasion by binding to TEAD4.^43^, ^44^ The Hippo pathway is composed of a kinase cascade, and the transcriptional coactivator YAP1 is up‐regulated in most human tumours.^45^, ^46^, ^47^ Therefore, the Hippo‐YAP1 signalling pathway and TEADs are potential targets for cancer therapy.^48^, ^49^, ^50^

In this study, we assessed whether the interaction between IRF2BP2 and VGLL4 affected the binding of YAP1 to TEAD4. The results of co‐immunoprecipitation using anti‐TEAD4 antibody indicated that IRF2BP2 knockdown increased the binding of TEAD4 to VGLL4 and decreased the binding of TEAD4 to YAP1, suggesting that IRF2BP2 enhanced the interaction between YAP1 and TEAD4, which could promote the transcription of genes downstream of YAP1.

CTGF is a critical gene downstream of YAP1. Activated YAP1 enters the nucleus, binds to TEAD4 and transcriptionally coactivates CTGF. It is an important oncogene related to cancer microenvironment and the progression of various cancers, including breast cancer,^20^ colorectal cancer,^51^ osteosarcoma,^52^ neuroblastoma 53 and GC.^15^ In this study, the knockdown of IRF2BP2 in SGC‐7901 and BGC‐823 cells decreased the mRNA and protein levels of CTGF, suggesting that IRF2BP2 regulated CTGF expression at the transcriptional level. Furthermore, the mRNA levels of IRF2BP2 and CTGF increased in clinical samples, and the up‐regulation of IRF2BP2 mRNA was positively linked with the up‐regulation of CTGF. CTGF mRNA expression was higher in patients with higher TNM stage, which is consistent with previous studies.^13^, ^14^, ^15^ Moreover, IRF2BP2 mRNA expression was positively associated with TNM stage and the depth of invasion, which is consistent with IHC results.

To further investigate whether this regulation depended on YAP1, IRF2BP2 was knocked down in the GC cell line MKN‐45, in which the expression of YAP1 was completely lost. However, IRF2BP2 knockdown did not affect CTGF expression at both the mRNA and protein levels, suggesting that IRF2BP2 regulates CTGF in a YAP1‐dependent manner.

It is of interest that a recent study demonstrated that VGLL4 bound to and stabilized IRF2BP2, leading to increased PD‐L1 expression and immune evasion through IRF2 inhibition.^23^ However, the IRF2BP2 sequence is highly conserved although in IRF‐2 protein‐deficient organisms, it implies the IRF2‐independent functions of IRF2BP2.^8^ The present results suggest that the binding of VGLL4 to IRF2BP2 may have an IRF‐2–independent function mediated by YAP1 in GC. In this respect, another study confirmed that IRF2BP2 presented IRF‐2–independent activities and affected the cell cycle, differentiation and apoptosis by negatively regulating the NFAT1‐mediated transactivation of NFAT‐responsive promoters.^9^ IRF2BP2 was also found to inhibit the p53‐mediated transactivation of the p21 and the BAX genes.^10^

MicroRNAs play important roles in transcriptional regulation and are frequently involved in human tumours.^30^, ^31^, ^32^ MicroRNA‐101‐3p acts as a tumour suppressor in GC by targeting the serum response factor directly and suppressing the proliferation and invasion of GC cells induced by HOX Transcript Antisense RNA (HOTAIR).^36^ Yan et al^54^ reported that lncRNA SNHG6 promoted cell proliferation and EMT by sponging miR‐101‐3p, leading to poor prognosis in GC. Luciferase activity assays were performed in present study to confirm that miR‐101‐3p targets 3′‐UTR of IRF2BP2 mRNA directly. The results demonstrated that up‐regulating miR‐101‐3p inhibited the expression of IRF2BP2 protein but not IRF2BP2 mRNA, indicating that miR‐101‐3p inhibited IRF2BP2 expression at a post‐transcriptional level. In SGC‐7901 and BGC‐823 cells, the overexpression of miR‐101‐3p inhibited CTGF expression at both mRNA and protein levels. However, in MKN‐45 cells, the lack of YAP1 blocked the regulation of miR‐101‐3p on CTGF.

Taken together, our findings indicate that the expression of IRF2BP2 is increased in GC, which is closely related to proliferation, migration and invasion, and contributes to poor prognosis in GC. The binding of IRF2BP2 to VGLL4 weakens the interaction between TEAD4 and VGLL4 and increases the binding of TEAD4 to YAP1, resulting in the transcriptional coactivation of CTGF expression. In addition, miR‐101‐3p inhibits IRF2BP2 expression by targeting the 3′‐UTR of IRF2BP2 mRNA, consequently suppressing CTGF expression in a YAP1‐dependent manner. We propose that the miR‐101‐3p‐IRF2BP2‐CTGF axis has a role in GC, and IRF2BP2 is a potential prognostic marker and therapeutic target in GC.

## CONFLICT OF INTEREST

The authors have no conflict of interest to declare.

## AUTHOR CONTRIBUTION

Yangyang Yao and Jun Deng wrote the manuscript. Jun Deng and Jianping Xiong designed the subject. Yangyang Yao, Yi Wang and Li Li conducted the cellular and animal experiments. Xiaojun Xiang, Junhe Li and Jun Chen worked on clinical information collection. Zhen Liu and Shanshan Huang participated in the data analysis. All authors read and approved the final manuscript.

## ETHICS APPROVAL AND CONSENT TO PARTICIPATE

All the patients agreed to participate in our study and provided written informed consent. All mice were cared in accordance with the National Guides for the Care and Use of Laboratory Animals and approved by the Institutional Animal Care and Use Committee of the First Affiliated Hospital of Nanchang University.

## Data Availability

The data sets supporting the conclusions of this article are included within the article.
