# Supplementary material for: Down‐regulation of interferon regulatory factor 2 binding protein 2 suppresses gastric cancer progression by negatively regulating connective tissue growth factor
Source: J Cell Mol Med. 2019 Sep 27;23(12):8076–89. doi: 10.1111/jcmm.14677 (PMC6851004; doi:10.1111/jcmm.14677)
Supplement: Supplementary file 1 [file JCMM-23-8076-s001.doc]

Table S1. Association of IRF2BP2 expression with survival rates

| Overall survival | OS | | |
| --- | --- | --- | --- |
|  | High expression | Low expression | Total |
| 1-year | 87.5% | 96.6% | 91.8% |
| 2-year | 62.5% | 82.8% | 72.1% |
| 5-year | 18.8% | 58.6% | 37.7% |
