# Supplementary material for: Down‐regulation of interferon regulatory factor 2 binding protein 2 suppresses gastric cancer progression by negatively regulating connective tissue growth factor
Source: J Cell Mol Med. 2019 Sep 27;23(12):8076–89. doi: 10.1111/jcmm.14677 (PMC6851004; doi:10.1111/jcmm.14677)
Supplement: Supplementary file 2 [file JCMM-23-8076-s002.doc]

**Table S2.** Clinicopathological characteristics of the gastric cancer patients ( M, male; F, female. ).

| **Patient No.** | **Age**  **(years)** | **Sex** | **TNM**  **classification** | **Clinical stage** | **Tumor size (cm^3^)** | **Histological grade**  **(differentiated)** |
| --- | --- | --- | --- | --- | --- | --- |
| 1 | 54 | F | T4aN1M0 | III | 6×4×3 | Low |
| 2 | 57 | F | T4aN0M0 | IIB | 9×9×3.5 | Low |
| 3 | 46 | M | T4aN1M0 | III | 11×9×3 | Moderate |
| 4 | 76 | F | T1N0M0 | I | 2×1.5×1.5 | High |
| 5 | 35 | M | T3N2M0 | III | 8×8×3.5 | Moderate |
| 6 | 66 | M | T2N2M0 | IIA | 4×2×1 | Moderate |
| 7 | 67 | M | T4aN1M0 | III | 9.5×7×4 | Low |
| 8 | 73 | F | T3N0M0 | IIB | 3×3×2 | Moderate |
| 9 | 44 | M | T4aN1M1 | III | 7×6×4 | Low |
| 10 | 54 | F | T2N0M0 | I | 2×2×1 | Moderate |
| 11 | 49 | M | T3N1M0 | III | 7.5×5×4 | Low |
| 12 | 61 | M | T3N2M0 | III | 8×5×4.5 | Low |
| 13 | 66 | F | T3N0M0 | IIB | 4×3×1 | Moderate |
| 14 | 61 | F | T4aN2M0 | III | 10×8.5×5 | Low |
| 15 | 51 | M | T1N2M0 | IIA | 3.5×2×1.5 | High |
| 16 | 52 | M | T2N1M0 | IIA | 3×2.5×1 | Moderate |
| 17 | 65 | F | T3N3M0 | III | 7×5.5×2.5 | Low |
| 18 | 54 | M | T3N1M0 | III | 5×5×3 | Low |
| 19 | 67 | M | T3N2M0 | III | 5×4.5×2 | Low |
| 20 | 65 | M | T4aN1M0 | III | 9×6.5×3 | Low |
| 21 | 50 | F | T3N2M0 | III | 4×3×4 | Moderate |
| 22 | 56 | M | T3N1M0 | III | 4×6×3 | Moderate |
| 23 | 48 | M | T4aN0M0 | IIB | 2×3×5 | Low |
| 24 | 61 | M | T3N3M0 | III | 3×6×3 | Moderate |
| 25 | 56 | F | T4aN2M0 | III | 6×3×4.5 | Low |
| 26 | 51 | F | T4aN0M0 | IIB | 2.5×4×3 | Moderate |
| 27 | 51 | M | T2N1M0 | IIA | 3.5×5×3 | Moderate |
| 28 | 49 | M | T4aN1M0 | III | 4×4×3.5 | Moderate |
| 29 | 58 | F | T2N0M0 | I | 1.5×1×2 | High |
| 30 | 63 | M | T4aN0M0 | IIB | 3.5×2×4 | Moderate |
| 31 | 70 | F | T4aN2M0 | III | 5×3×3 | Low |
| 32 | 60 | F | T3N1M0 | III | 4×4.5×3 | Moderate |
| 33 | 60 | M | T2N3M0 | IIA | 2×2×1.5 | Moderate |
| 34 | 56 | F | T2N1M0 | IIA | 4×1.5×3 | Low |
| 35 | 43 | M | T1N1M0 | IIA | 3×1.5×2 | Moderate |
| 36 | 44 | M | T4aN0M0 | IIB | 2.5×2×2 | Moderate |
| 37 | 59 | M | T3N1M0 | III | 5×3.5×3 | Low |
| 38 | 57 | M | T3N3M0 | III | 4×2×3 | Low |
| 39 | 61 | F | T3N3M0 | III | 3×2.5×3.5 | Moderate |
| 40 | 39 | M | T1N0M0 | I | 1×1×1.5 | High |
